# Integrative Analysis of Neutrophil-Associated Genes Reveals Prognostic Significance and Immune Microenvironment Modulation in Cervical Cancer

**DOI:** 10.3390/biomedicines13061348

**Published:** 2025-05-30

**Authors:** Ting Hu, Haijing Wu, Xinghan Cheng, Haoyue Gao, Min Yang

**Affiliations:** 1 Department of Gynaecological Oncology, Sichuan Clinical Research Center for Cancer, Sichuan Cancer Hospital & Institute, Sichuan Cancer Center, University of Electronic Science and Technology of China, Chengdu 610041, China; huting@scszlyy.org.cn (T.H.); wuhaijing128@hotmail.com (H.W.); chengxinghan456@163.com (X.C.); 2 Department of Geriatrics, Women and Children, School of Nursing, Chengdu Medical College, Chengdu 610500, China; corkiagao@msn.com

**Keywords:** cervical cancer, immune microenvironment, neutrophil-associated genes, prognostic model, SEMA6B

## Abstract

**Background:** Tumour-associated neutrophils play an important role in tumour progression and immunomodulation. However, the prognostic significance and immunological implications of neutrophil-associated genes (NAGS) in cervical cancer remain poorly defined. **Methods:** We analyzed neutrophil infiltration and its correlation with gene expression in TCGA cervical cancer data using immune deconvolution. NAGS were identified via correlation and enrichment analysis. A prognostic model was constructed using Cox and LASSO regression and validated in the GSE30759 cohort. Kaplan–Meier analysis, ROC curves, and multivariate Cox regression were used to assess prognostic performance. The model’s association with the tumor immune microenvironment and immunotherapy response was further analyzed. The expression pattern of SEMA6B was explored using cell lines, clinical subgroups, and human protein profiles, and its immunological relevance was evaluated using multiple immune infiltration algorithms. **Results:** Twelve genes were identified as significantly correlated with neutrophil infiltration and enriched in immune-related pathways such as chemotaxis, neutrophil degranulation, and PI3K-AKT signaling. Further NAGS models were developed based on key genes. High-risk patients exhibited an immunosuppressive tumor microenvironment, elevated TIDE scores, and lower predicted responsiveness to immunotherapy. SEMA6B was significantly downregulated in the tumour group but may be reactivated during metastasis. High expression of SEMA6B was associated with poorer prognostic features and immune evasion. **Conclusions:** We developed a NAGS signature that may inform prognosis and immune microenvironment status in cervical cancer. These findings suggest the potential clinical utility of NAGs-based models in guiding immunotherapy strategies. Moreover, SEMA6B may serve as a promising immunological and prognostic biomarker, pending further mechanistic validation.

## 1. Introduction

Cervical cancer continues to rank among the most prevalent malignancies affecting the female reproductive system [1,2,3]. Although progress in vaccination and early detection has been achieved, outcomes for patients with advanced or recurrent disease remain unsatisfactory [4,5,6]. Traditional prognostic indicators—such as FIGO stage, histological grade, and lymph node status—are widely used in clinical settings; however, they often fail to fully capture tumor heterogeneity or reflect the immune landscape of the tumor. As a result, their predictive value for immunotherapy responsiveness remains limited. In contrast, molecular signatures derived from tumor transcriptomes offer more nuanced stratification and may facilitate precision oncology approaches. This highlights the pressing need for novel biomarkers that not only forecast clinical prognosis but also assist in optimizing immunotherapy strategies [7,8].

The tumor microenvironment (TME) has increasingly been recognized as a key player in influencing tumor behavior and treatment efficacy [9,10]. Neutrophils, which constitute an essential part of innate immunity, have been implicated in modulating the TME. Tumor-associated neutrophils (TANs) contribute to diverse biological functions including neovascularization, tumor cell invasion, and immune evasion [11,12]. Depending on the tumor type and microenvironmental cues, TANs may exhibit either tumor-promoting or tumor-inhibiting properties [13,14,15].

Emerging evidence suggests that neutrophil infiltration is associated with poor prognosis and advanced stages of cervical cancer [16,17]. In non-small cell lung cancer and gastric cancer, neutrophil-rich tumors have been shown to exhibit increased angiogenesis, higher rates of metastasis, and reduced T cell infiltration—features that contribute to immune evasion and resistance to therapy. Neutrophil-associated genes (NAGS) have been identified as critical regulators of neutrophil recruitment, activation, and function within the TME [18,19]. These genes are linked to key biological processes, including chemotaxis, cytokine-mediated signaling, and neutrophil degranulation [20,21]. Studies in lung, gastric, and colorectal cancers have demonstrated that NAG-based models can serve as prognostic markers and guide therapeutic interventions [22,23,24].

However, a comprehensive investigation of neutrophil-associated gene signatures in cervical cancer integrating large-scale transcriptomic data with immune deconvolution and clinical outcome modeling has not been previously reported. This gap may be attributed to the historical underrepresentation of cervical cancer in immunogenomic research and the absence of neutrophil-focused frameworks in existing cervical cancer prognostic models.

**Figure 1:**
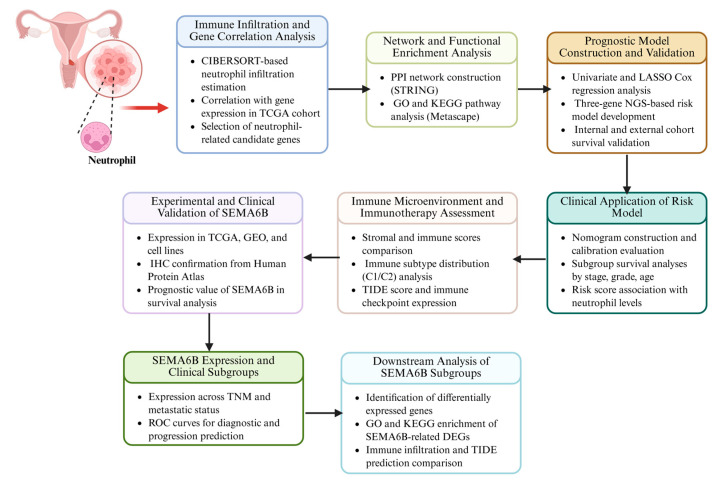
Analytical workflow for neutrophil-related gene investigation in cervical cancer.

In this study, we systematically analyzed the role of neutrophils in cervical cancer. We identified key NAGS associated with neutrophil infiltration and constructed a prognostic model to evaluate their clinical significance. Furthermore, we investigated how the NAGS risk score influences the TME, including its impact on immune infiltration and immunotherapy outcomes. By addressing a previously unexplored axis of tumor-immune interaction, our findings provide new insights into the immunobiology of cervical cancer and offer translational potential for improving risk stratification and guiding immune-based therapies. The flow chart of this study is shown in Figure 1.

## 2. Materials and Methods

### 2.1. Data Collection and Preprocessing

Transcriptomic datasets and corresponding clinical annotations for cervical cancer patients were retrieved from The Cancer Genome Atlas (TCGA, https://portal.gdc.cancer.gov/) [25,26] and the GEO dataset GSE30759 (https://www.ncbi.nlm.nih.gov/geo/) [27,28]. Samples were included if they met the following criteria: (1) available gene expression data; (2) complete overall survival (OS) information; and (3) accompanying clinical information including stage and grade. Samples were excluded if they had missing survival outcomes, incomplete expression data, or poor-quality annotations.

This study was retrospective in design, and all data analyzed were quantitative in nature, derived from publicly available transcriptomic and clinical datasets. Only samples with complete overall survival data and gene expression profiles were included. For downstream analysis, gene expression values were transformed using log2(TPM + 1).

### 2.2. Tumor Microenvironment Analysis

To assess the tumour immune microenvironment, we applied the CIBERSORT algorithm to estimate the immune cell types in each sample and clustered the immune cell infiltration patterns using the ‘ConsensusClusterPlus’ R package [29,30]. Stromal and immune scores and tumour purity were calculated using the ESTIMATE algorithm. The TIMER database (http://timer.cistrome.org) was used as a gene expression correlation analysis to assess neutrophil infiltration.

Potential biases include reliance on computational deconvolution methods (e.g., CIBERSORT), which may not capture spatial immune heterogeneity or account for tumor purity variations.

### 2.3. Identification of Neutrophil-Associated Genes

To identify NAGS relevant to cervical cancer, we first estimated the abundance of neutrophil infiltration in each sample using the CIBERSORT algorithm based on bulk RNA-seq data from the TCGA cohort [23,31,32]. We then calculated Pearson correlation coefficients between the expression levels of all protein-coding genes and the estimated neutrophil infiltration levels. Genes were retained as neutrophil-associated candidates if they met both of the following criteria: an absolute Pearson correlation coefficient (|R|) greater than 0.3 and a *p*-value less than 0.001. The |R| > 0.3 threshold was chosen to ensure a moderate-to-strong correlation, sufficient to reflect a meaningful biological association, while minimizing the inclusion of noise. The *p*-value < 0.001 cut-off provides statistical stringency and reduces the false discovery rate given the large number of correlation tests performed. These thresholds are consistent with previous immune correlation studies and help ensure robustness and reproducibility.

The STRING database and Metascape database were used to perform PPI network and functional enrichment analysis on key NAGS [33].

### 2.4. Construction and Validation of the Prognostic Model

A neutrophil-associated prognostic model was developed by first performing univariate Cox regression analysis to identify NAGS significantly associated with overall survival. LASSO regression was subsequently applied to eliminate redundant variables and prevent model overfitting. A final prognostic signature was established using multivariate Cox regression. A risk score (RS) was calculated for each patient as the weighted sum of gene expression levels: RS = ∑ (expression × coefficient) for the selected genes (SEMA6B, CSF2RB, and IRF4). External validation was conducted using the GEO dataset GSE30759. A nomogram integrating risk score and clinical features was constructed, and calibration plots were used to evaluate its predictive accuracy.

### 2.5. Gene Expression Validation and Cell Experiments

HeLa and CaSki cervical cancer cell lines, along with the normal cervical epithelial cell line ECT1, were cultured in DMEM supplemented with 10% FBS under standard conditions. The PCR procedure was described in a previously published article [34,35]. Primer sequences were as follows: GAPDH, forward 5′-GGAGCGAGATCCCTCCAAAAT-3′, reverse 5′-GGCTGTTGTCATACTTCTCATGG-3′; SEMA6B, forward 5′-GTCGGAGACAACATCAGCGGTA-3′, reverse 5′-GCATCAATGGCTAGGAAGTCGG-3′. In addition, we analyzed the protein expression of NAGS using the Human Protein Atlas (http://www.proteinatlas.org) and obtained representative immunohistochemistry images to compare expression levels between tumor and normal tissues.

### 2.6. Immunotherapy Data Analysis

We evaluated potential responses to immunotherapy using immunophenoscore (IPS) and TIDE data obtained from TCIA (https://tcia.at/home). As IPS is a well-established predictor of CTLA-4 and PD-1 checkpoint inhibitor responsiveness, we downloaded the corresponding IPS files from TCIA. We then compared IPS values, TIDE scores, and immune checkpoint gene expression levels between subgroups to assess differences in predicted immunotherapy sensitivity [36,37].

### 2.7. Statistical Analysis

All statistical procedures were conducted using R software (version 4.1.2). For comparisons between two groups, either the Student’s *t*-test or Wilcoxon test was applied. When analyzing more than two groups, one-way ANOVA or the Kruskal–Wallis test was used, depending on data distribution. A two-tailed *p*-value below 0.05 was considered indicative of statistical significance.

## 3. Results

### 3.1. Identification of Neutrophil-Associated Genes and Prognostic Signatures in Cervical Cancer

**Figure 2:**
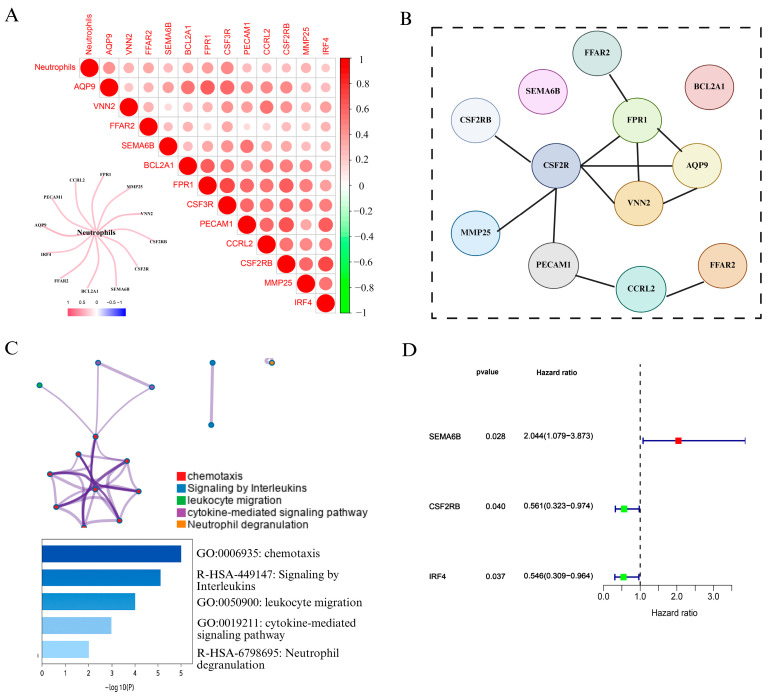
Identification and characterization of NAGS in cervical cancer. (**A**) Correlation analysis between neutrophil infiltration and gene expression in the TCGA cervical cancer dataset. The heatmap displays Pearson correlation coefficients between neutrophils and 12 candidate genes. The color scale indicates the correlation strength (red = positive, green = negative), and a network plot illustrates the strength of correlation with neutrophils. (**B**) PPI network constructed using the STRING database, revealing potential interactions among the 12 neutrophil-associated genes. (**C**) Functional enrichment analysis of NAGS using Metascape. Enriched biological pathways include chemotaxis, leukocyte migration, cytokine-mediated signaling pathway, interleukin signaling, and neutrophil degranulation. The bar plot shows −log10(*P*) values for the top enriched terms. (**D**) Univariate Cox regression analysis of NAGS in the TCGA cohort.

To explore the role of neutrophils in cervical cancer, we first analyzed immune cell infiltration using the CIBERSORT algorithm based on TCGA cervical cancer transcriptomic data. As shown in Figure 2A and Figure S1, correlation analysis revealed that 12 genes, including FPR1, VNN2, PECAM1, CCR2, and CSF3R, were strongly positively associated with neutrophil infiltration. We visualized these results using a heatmap and correlation matrix, where red intensity indicated stronger positive correlations, while green denoted negative correlations. We also constructe a network diagram to further illustrated the strength of associations between neutrophils and candidate genes, with thicker red lines indicating stronger interactions.

To explore potential interactions among the NAGS set, a protein interaction network was generated using STRING (Figure 2B). Subsequently, Metascape was employed to conduct pathway and functional enrichment analysis of the identified genes. As illustrated in Figure 2C, the gene set was predominantly involved in immune-related biological processes, such as chemotactic activity, leukocyte trafficking, interleukin signaling, and pathways mediated by cytokines. Notably, neutrophil degranulation emerged as the most significantly enriched term, underscoring the immunological role of these genes in neutrophil function.

Following the identification of NAGS, we next evaluated their prognostic value in cervical cancer. Univariate Cox regression analysis in the TCGA cohort revealed that three genes—SEMA6B, CSF2RB, and IRF4—were significantly associated with overall survival (*p* < 0.05). Among these, SEMA6B was identified as a risk factor (HR > 1), whereas CSF2RB and IRF4 acted as protective factors (HR < 1).

### 3.2. Construction and Validation of a Prognostic Risk Model Based on Neutrophil-Associated Genes

**Figure 3:**
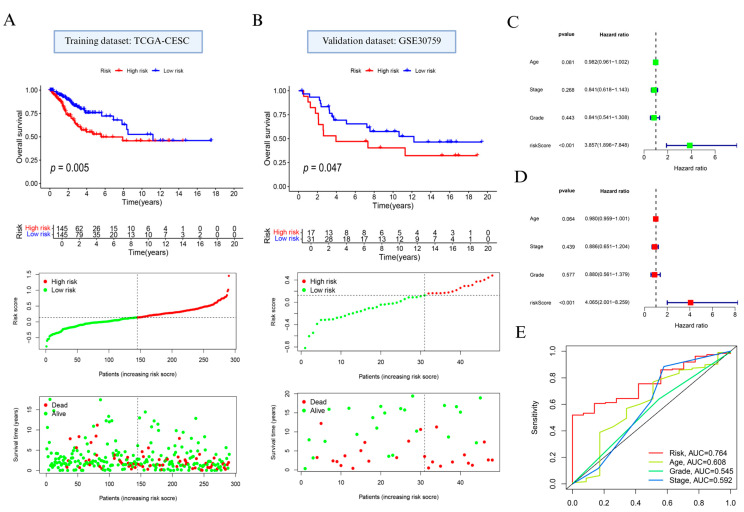
Development and evaluation of the neutrophil-associated gene signature in cervical cancer. (**A**) Overall survival comparison, gene signature-based risk stratification, and survival outcome visualization in the TCGA training dataset. (**B**) Validation of survival discrimination and risk pattern in the GSE30759 cohort. (**C**) Univariate and (**D**) multivariate Cox proportional hazards models used to assess the independent prognostic contribution of the constructed risk model. (**E**) Receiver operating characteristic curve analysis comparing the predictive performance of the risk signature with clinical variables including patient age, tumor stage, and histological grade.

Based on prognostically relevant NAGS, we constructed a prognostic model using LASSO regression (Supplementary Figure S2A,B; Supplementary Table S1). Based on the median value of the risk score, the patients with cervical cancer were classified into high and low subgroups. Prognostic analysis indicated that individuals in the high-risk group exhibited significantly reduced OS compared to those in the low-risk group (*p* = 0.005; Figure 3A, top). Additionally, distribution of risk scores and survival status (Figure 3A, bottom) showed that elevated risk scores were linked to increased mortality.

Model performance was further confirmed in the external GSE30759 dataset. In alignment with findings from the training cohort, the high-risk subgroup showed poorer OS (*p* = 0.047; Figure 3B, top), and the survival landscape analysis reiterated the unfavorable prognosis linked to higher NAGS-derived scores (Figure 3B, bottom).

In addition, the results of Cox regression analysis showed that RS had the potential to be a risk indicator for cervical cancer (*p* < 0.001; Figure 3C,D). We further examined the model’s discriminatory ability using time-dependent ROC analysis. As shown in Figure 3E, the AUC for the NAGS score reached 0.764, surpassing age (0.608), stage (0.592), and grade (0.545).

To further assess the model’s robustness and reduce potential overfitting risk, we performed 5-fold cross-validation within the TCGA cohort. As shown in Supplementary Figure S3, the cross-validation results demonstrated consistent predictive performance, with an average C-index of 0.741 across folds, supporting the stability and generalizability of the model.

### 3.3. Clinical and Immunological Characterization of the NAGS-Based Risk Model

**Figure 4:**
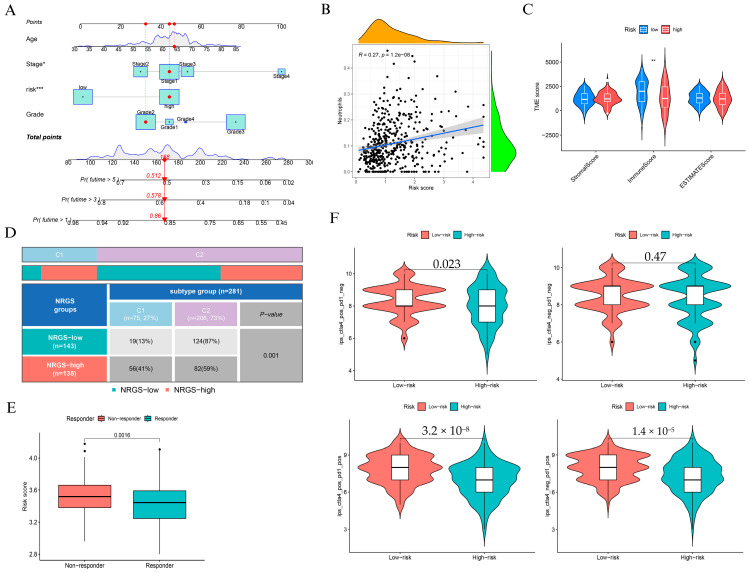
Clinical application and immune characterization of the NAGS-based risk model. (**A**) Nomogram integrating risk score, age, stage, and grade to predict 1-, 3-, and 5-year overall survival. (**B**) Correlation between NRGS and neutrophil infiltration. (**C**) Comparison of StromalScore, ImmuneScore, and ESTIMATEScore between low- and high-risk groups. (**D**) Distribution of NRGS risk groups across immune subtypes “Wound Healing” (C1) and “IFN-γ Dominant” (C2) (*p* = 0.001). (**E**) NRGS risk score in immunotherapy responders versus non-responders (*p* = 0.0016). (**F**) Immune scores across treatments: CTLA4+ PD1-, CTLA4- PD1-, CTLA4+ PD1+, and CTLA4- PD1+, comparing low- and high-risk groups. * *p* < 0.05, ** *p* < 0.01, and *** *p* < 0.001.

To assess the clinical utility of the NAGS-based risk score, we constructed a nomogram incorporating age, stage, grade, and risk score to predict 1-, 3-, and 5-year overall survival (Figure 4A). Calibration analysis confirmed strong concordance between predicted and observed outcomes, particularly at 1 and 3 years (Supplementary Figure S4). Kaplan-Meier survival analyses (Supplementary Figure S5) consistently showed that high-risk patients had significantly poorer survival outcomes across different subgroups of grade, age, and stage (*p* = 0.027, *p* = 0.028, and *p* = 0.021, respectively).

We observed a moderate but statistically significant association between the risk score and neutrophil infiltration (R = 0.27, *p* = 1.2 × 10^−8^; Figure 4B), suggesting that elevated NAGS scores may reflect a pro-inflammatory tumor immune landscape. However, StromalScore and ImmuneScore were significantly lower in the high-risk group (Figure 4C), suggesting an overall immunosuppressive phenotype. Immune subtype distribution differed markedly between groups: high-risk patients were enriched in the wound healing subtype (C1), while low-risk patients predominantly belonged to the IFN-γ dominant subtype (C2) (*p* = 0.001; Figure 4D).

In terms of immunotherapy response, we found that non-responders exhibited significantly higher risk scores than responders (*p* = 0.0016; Figure 4E). Moreover, PD-L1 (CD274) and CTLA4 were significantly upregulated in the high-risk group (Supplementary Figure S6), reinforcing the presence of immune evasion features. We further compared immune scores under different checkpoint blockade regimens and found that low-risk patients consistently demonstrated higher immune scores, particularly under PD-1 monotherapy and combined PD-1/CTLA-4 blockade (Figure 4F).

### 3.4. Expression Validation and Clinical Significance of SEMA6B in Cervical Cancer

**Figure 5:**
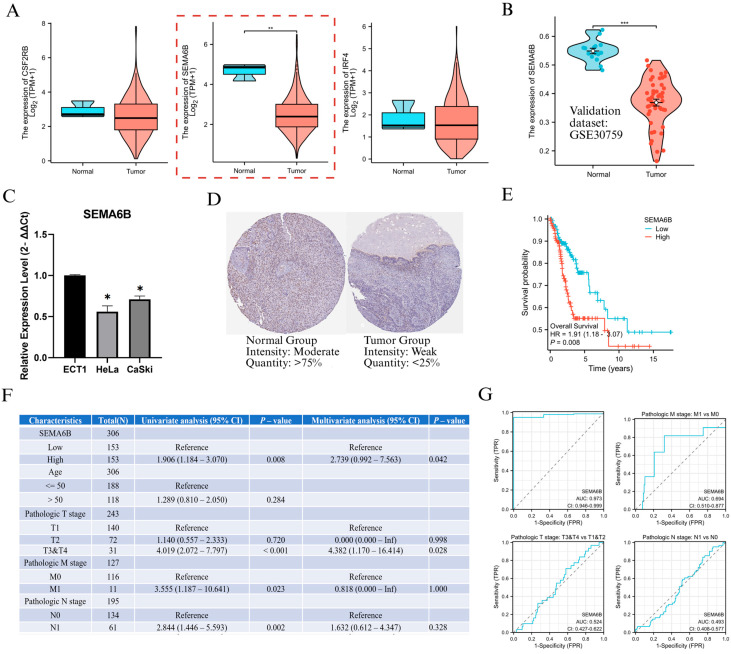
Expression validation and clinical relevance of SEMA6B in cervical cancer. (**A**) Expression levels of CSF2RB, SEMA6B, and IRF4 in normal and tumor tissues from the TCGA dataset. (**B**) Validation of SEMA6B downregulation in the GSE30759 dataset. (**C**) qRT-PCR analysis of SEMA6B expression in normal and cervical cancer cell lines. (**D**) Immunohistochemical staining of SEMA6B in normal and tumor tissues from the HPA. (**E**) Kaplan–Meier survival analysis based on SEMA6B expression in TCGA cohort. (**F**) Univariate and multivariate Cox regression analysis of SEMA6B and clinical parameters. (**G**) ROC curves evaluating the predictive performance of SEMA6B across clinical subgroups. Note: Although SEMA6B is overall downregulated in cervical cancer, Cox regression analysis (Figure 5F) shows that higher expression is associated with poorer prognosis and may be modestly upregulated in metastatic subgroups (M1 vs. M0), suggesting a context-dependent reactivation in advanced disease. *, *p* < 0.05; **, *p* < 0.01; and ***, *p* < 0.001.

To investigate the biological relevance of genes within the NAGS model, we first analyzed the expression levels of CSF2RB, SEMA6B, and IRF4 in the TCGA cervical cancer dataset. Among them, SEMA6B was significantly downregulated in tumor tissues compared to normal samples (*p* < 0.01; Figure 5A). This result was further validated in the GSE30759 dataset, where SEMA6B expression remained markedly lower in tumor tissues (*p* < 0.001; Figure 5B). In Furthermore, PCR results showed that SEMA6B expression was significantly reduced in cervical cancer cell lines compared to normal cell lines (Figure 5C). In protein level, expression was weaker or absent in tumour tissues compared to normal tissues, further supporting the transcriptomic findings (Figure 5D). Prognostic analysis showed that patients with high SEMA6B expression had significantly shorter overall survival (HR = 1.901, *p* = 0.008; Figure 5E). Both univariate and multivariate Cox regression confirmed SEMA6B as an independent prognostic factor (HR = 2.739; Figure 5F).

We also evaluated SEMA6B expression across various clinical subgroups (Supplementary Figure S7). Although expression was consistently downregulated in tumors across all clinical stages (*p* < 0.05), we observed an upward trend in advanced disease, particularly in metastatic cases (M1 vs. M0, *p* < 0.05), suggesting a potential reactivation of SEMA6B under metastatic conditions. ROC analysis showed that SEMA6B had a good predictive ability in distinguishing tumour from normal tissue and in identifying metastatic potential (Figure 5G).

### 3.5. Differential Expression and Functional Enrichment Analysis Between SEMA6B Expression Subgroups

**Figure 6:**
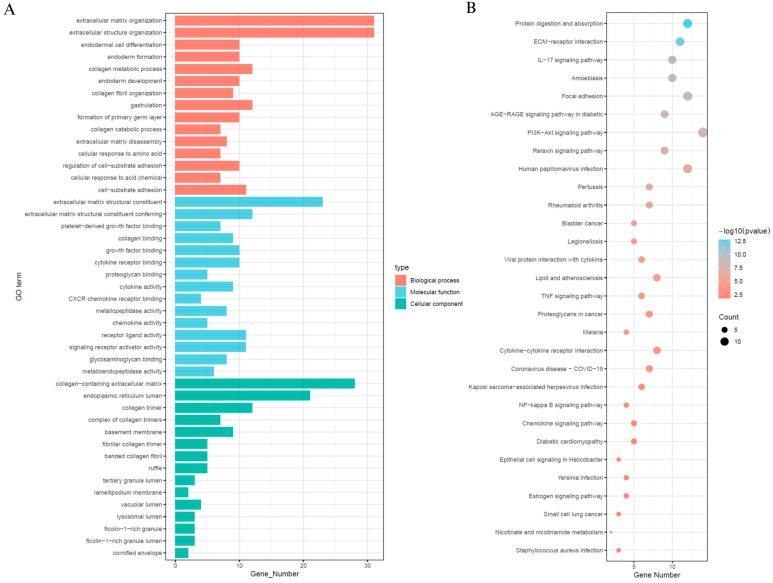
Functional enrichment analysis of differentially expressed genes between high- and low-SEMA6B expression groups. (**A**) Gene Ontology (GO) enrichment analysis of DEGs, including biological processes (red), molecular functions (blue), and cellular components (green). (**B**) KEGG pathway enrichment analysis of DEGs. Dot size indicates gene count, and color represents significance level (−log10(*p*-value)).

In order to explore the signalling mechanisms that may be associated with SEMA6B in cervical cancer, we performed differential gene expression analyses comparing SEMA6B high and low expression subgroups, from which we identified 71 significant DEGs (Supplementary Figure S8). Subsequent functional annotation was performed using GO and the KEGG to investigate the biological characteristics of these differentially expressed genes (Figure 6). Gene Ontology enrichment indicated that these genes were primarily involved in extracellular matrix organization, collagen biosynthesis, cytokine receptor activity, and cell-substrate adhesion (Figure 6A). KEGG and GO analysis further revealed enrichment in phosphatidylinositol 3-kinase–protein kinase B signaling pathway, interleukin-17 signaling pathway, focal adhesion, and extracellular matrix–receptor (Figure 6B).

These pathways are known to play critical roles in cervical cancer pathogenesis. In particular, the PI3K–Akt signaling pathway is frequently activated in HPV-associated cervical tumors and is implicated in promoting cell proliferation, survival, and angiogenesis [38,39]. Likewise, IL-17 signaling has been associated with chronic inflammation and immune dysregulation in the cervical tumor microenvironment [40,41]. The observed enrichment in extracellular matrix–related processes and focal adhesion suggests that SEMA6B may contribute to epithelial–mesenchymal transition (EMT), a key mechanism in cervical cancer invasion and metastasis.

### 3.6. Association Between SEMA6B Expression and Tumor Immune Microenvironment

**Figure 7:**
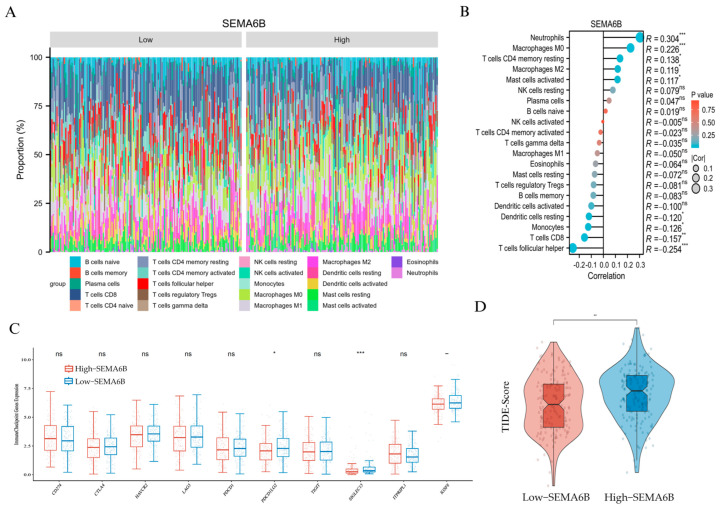
Immunological characteristics and immunotherapy prediction associated with SEMA6B expression. (**A**) Relative proportions of 22 immune cell types in the high- and low-SEMA6B expression groups estimated by CIBERSORT. (**B**) Spearman correlation between SEMA6B expression and immune cell infiltration. (**C**) Expression levels of selected immune checkpoint genes between high- and low-SEMA6B groups. (**D**) Comparison of TIDE scores between high- and low-SEMA6B expression groups; higher TIDE scores indicate reduced responsiveness to immunotherapy. *, *p* < 0.05; **, *p* < 0.01; and ***, *p* < 0.001.

The immune composition profile revealed distinct distributions of immune cells between the two groups (Figure 7A). Correlation analysis demonstrated that SEMA6B expression was positively associated with neutrophils (R = 0.304), macrophages M0 (R = 0.226), and resting CD4+ memory T cells (R = 0.138), but negatively correlated with CD8+ T cells (R = −0.157) and follicular helper T cells (R = −0.254), suggesting a potentially immunosuppressive phenotype (Figure 7B).

To further validate these findings, we applied the single-sample Gene Set Enrichment Analysis (ssGSEA) algorithm to assess the immune cell landscape in relation to SEMA6B expression. As shown in Supplementary Figure S9, SEMA6B was strongly positively correlated with neutrophils (R = 0.448), mast cells, eosinophils, and macrophages, confirming an innate immune-dominated microenvironment. In contrast, the association with adaptive immune subsets, including CD8^+^ T cells, T helper cells, and regulatory T cells, was weak or negative. These results were consistent with the CIBERSORT-based observations and provide orthogonal computational evidence supporting the role of SEMA6B in shaping a myeloid-enriched, immunosuppressive tumor microenvironment.

We then compared immune checkpoint gene expression between groups. Notably, high-SEMA6B expression was associated with significantly lower expression of PDCD1LG2 and SIGLEC15, while no significant differences were observed in other classical checkpoint molecules such as PDCD1, CTLA4, or LAG3 (Figure 7C).

Finally, we evaluated potential response to immunotherapy using TIDE scoring. The high-SEMA6B group exhibited significantly higher TIDE scores (*p* < 0.01), indicating a higher likelihood of immune escape and reduced immunotherapy responsiveness (Figure 7D). These findings suggest that SEMA6B may serve as a marker of immune suppression and unfavorable immunotherapy response in cervical cancer.

## 4. Discussion

Neutrophils have emerged as dynamic regulators within the tumor microenvironment, capable of exerting both pro-tumor and anti-tumor effects depending on the context [42]. In cervical cancer, tumor-associated neutrophils have been linked to disease progression, metastasis, and poor prognosis [18,19]. Mechanistically, neutrophils may promote tumor invasion and immune evasion through the release of cytokines, chemokines, matrix metalloproteinases, and reactive oxygen species [43,44,45]. However, the gene-level determinants underlying neutrophil recruitment and function in cervical cancer remain incompletely defined.

This study presents a comprehensive analysis of neutrophil-associated gene signatures in cervical cancer, aiming to elucidate their prognostic significance and potential influence on the tumor immune microenvironment. By integrating transcriptomic and clinical data from TCGA and validating the results in an independent cohort, we identified a set of genes correlated with neutrophil infiltration and developed a risk model that may inform patient prognosis, immune context, and potential therapeutic responsiveness. These findings offer insights into the immunological heterogeneity of cervical cancer and provide a foundation for the exploration of immune-related biomarkers.

In this study, we identified twelve NAGS that showed significant positive correlation with neutrophil infiltration in cervical cancer tissues. These genes were enriched in pathways related to extracellular matrix remodeling, leukocyte chemotaxis, cytokine–cytokine receptor interactions, neutrophil degranulation, and IL-17 signaling, suggesting that NAGS may participate in both immune activation and immune regulation [46,47,48]. Notably, enrichment of the PI3K-AKT signaling pathway and focal adhesion pathways may indicate involvement of NAGS in tumor proliferation, angiogenesis, and immune suppression [49,50].

We subsequently constructed a NAGS prognostic model based on SEMA6B, CSF2RB, and IRF4. This risk score demonstrated strong predictive capacity for overall survival in both the training and validation cohorts. High-risk patients were characterized by increased neutrophil infiltration, lower stromal and immune scores, and higher likelihood of immune exclusion or dysfunction. These features were accompanied by elevated expression of immune checkpoint genes and increased TIDE scores, suggesting that the model may reflect not only clinical prognosis but also the immunological profile of the tumor.

Among the three genes, SEMA6B emerged as a particularly interesting candidate for further study. It was significantly downregulated in cervical cancer tissues and cell lines, but higher expression was paradoxically associated with worse overall survival and immunosuppressive signatures, including reduced CD8^+^ T cell infiltration and elevated TIDE scores. In metastatic patients, a mild upregulation of SEMA6B was observed, suggesting that its expression may be reactivated in advanced disease stages or specific immunosuppressive microenvironments. This paradoxical pattern suggests that SEMA6B may play a context-dependent role in cervical cancer progression. One plausible explanation is that SEMA6B is downregulated during early tumor development but re-expressed in later stages under immune-selective pressure, contributing to immune escape and tumor dissemination.

In particular, SEMA6B may facilitate tumor progression by modulating semaphorin–plexin signaling, which is known to influence immune suppression, angiogenesis, and cell motility. The enrichment of SEMA6B-related genes in pathways such as PI3K-Akt, IL-17, and extracellular matrix remodeling further supports its potential role in EMT, a hallmark of tumor invasion and metastasis [38,39,41,51]. In this context, high SEMA6B expression may reflect an aggressive tumor phenotype associated with stromal remodeling and immune evasion.

Furthermore, semaphorin family members, including SEMA6B, have been shown to exhibit pleiotropic and even opposing effects depending on cellular context and microenvironmental cues in various malignancies [52,53,54]. These results indicate that SEMA6B may serve as a context-dependent marker with both diagnostic and prognostic value, although the underlying mechanisms require further investigation.

In support of our findings, recent studies have also highlighted the prognostic and therapeutic value of NAGS in other cancer types. For instance, in gastric cancer, neutrophil-related signatures were shown to define molecular subtypes with distinct epithelial-neutrophil interactions and therapeutic vulnerabilities [55]. Similarly, Tang et al. developed a neutrophil-centered model in gastric cancer based on single-cell and bulk transcriptomics, which revealed poor prognosis associated with neutrophil-enriched tumors and identified QKI as a key splicing regulator linked to neutrophil function and drug resistance [23]. In colorectal cancer, Yang et al. established a prognostic risk score (PRS) based on NAGs that not only predicted survival but also correlated with immune suppression and response to immunotherapy, and showed utility as a potential “pan-cancer” immunotherapy biomarker [24]. These findings collectively suggest that neutrophil-associated signatures are clinically relevant across multiple tumor types and may represent a universal axis of tumor-immune regulation.

Our study contributes to this growing body of evidence by extending NAGs-based modeling to cervical cancer and demonstrating its value in predicting prognosis and immune response potential.

Despite the robustness of our analysis, several limitations should be acknowledged. First, the study is primarily based on retrospective public datasets, which may introduce inherent biases and confounding factors. Second, the findings, although validated across cohorts and supported by in vitro expression data, are largely correlative and lack mechanistic validation. Third, immune infiltration was inferred through computational algorithms, which may not fully reflect the spatial complexity and functional status of tumor-infiltrating immune cells.

Therefore, future research will aim to experimentally validate the biological functions of NAGS and SEMA6B in cervical cancer. This includes conducting gene expression assays, gene manipulation experiments, and spatial immune profiling. Moreover, translating the NAGS-based risk model into clinical application will require further assessment of its predictive utility in prospective cohorts and integration with immunotherapeutic strategies.

In conclusion, we developed a neutrophil-associated gene signature that may provide prognostic information and reflect the immune microenvironment and therapeutic response in cervical cancer. Moreover, SEMA6B is identified as a potential prognostic indicator that is associated with both adverse clinical outcomes and immunosuppressive features in cervical cancer.

## Data Availability

The original contributions presented in the study are included in the article/Supplementary Material. Further inquiries can be directed to the corresponding authors.

## Supplementary Materials

The following supporting information can be downloaded at: https://www.mdpi.com/article/10.3390/biomedicines13061348/s1, Figure S1: Identification of Neutrophil-Associated Genes, Figure S2: LASSO regression for prognostic model construction, Figure S3: 5-Fold Cross-Validation of the NAGS-Based Prognostic Model in the TCGA Cohort, Figure S4: Calibration plot of the nomogram for predicting overall survival, Figure S5: Stratified survival analysis of the NAGS-based risk model, Figure S6. Expression of immune checkpoint molecules CD274 (PD-L1) and CTLA4 between risk groups, Figure S7. SEMA6B expression across pathological stages in cervical cancer, Figure S8: Differentially expressed genes (DEGs) between high- and low-SEMA6B expression groups; Table S1: Hazard Ratios and Confidence Intervals for Prognostic Genes Included in the NAGS-Based Risk Model.
