# Supplementary material for: Integrative Analysis of Neutrophil-Associated Genes Reveals Prognostic Significance and Immune Microenvironment Modulation in Cervical Cancer
Source: Biomedicines. 2025 May 30;13(6):1348. doi: 10.3390/biomedicines13061348 (PMC12189475; doi:10.3390/biomedicines13061348)
Supplement: Supplementary file 1 [file biomedicines-13-01348-s001.zip › biomedicines-3612080-supplementary.docx]

**Supplementary Materials:**


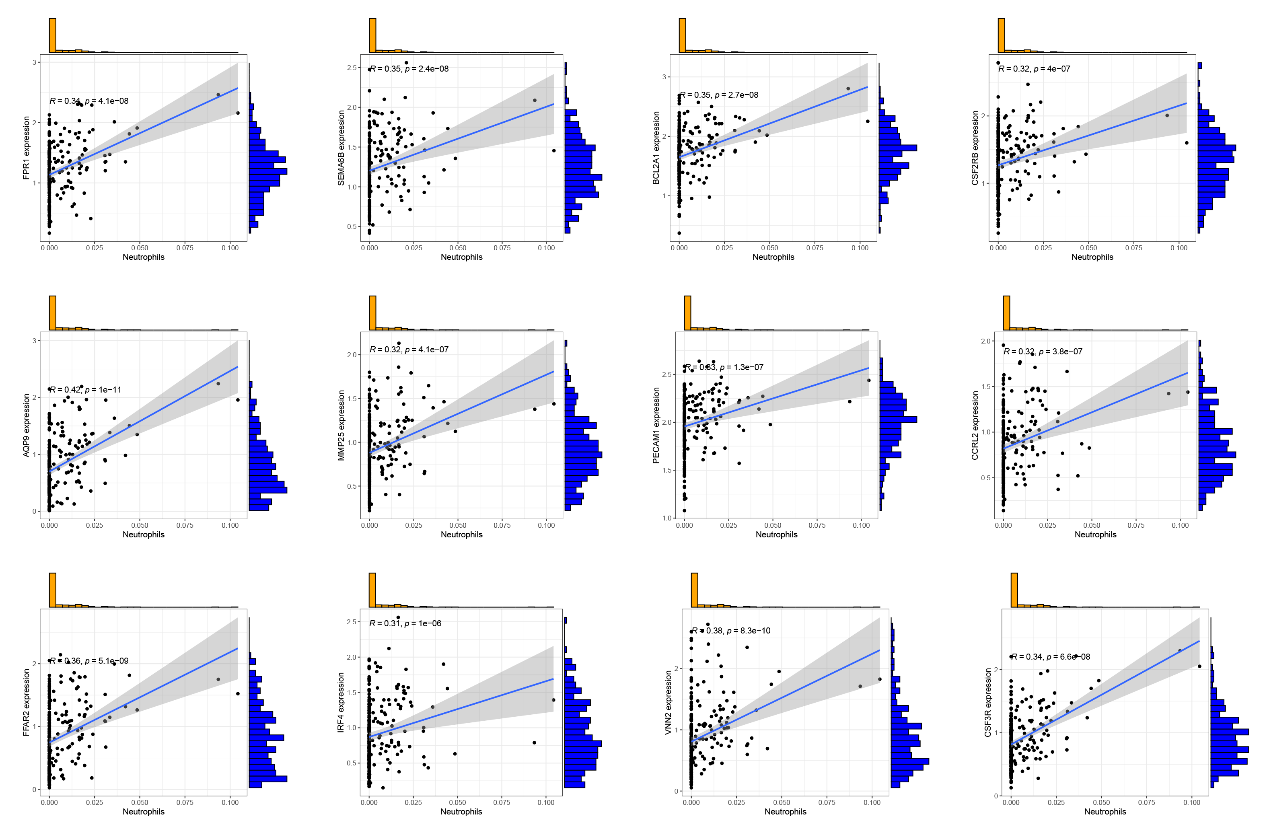


**Figure S1. Identification of Neutrophil-Associated Genes.**

Scatter plots showing the correlation between neutrophil infiltration and the expression of 12 genes (FPR1, VNN2, PECAM1, CCR2, CSF3R, MMP25, IRF4, BCL2A1, SEMA6B, AQP9, FFAR2, CSF2RB).


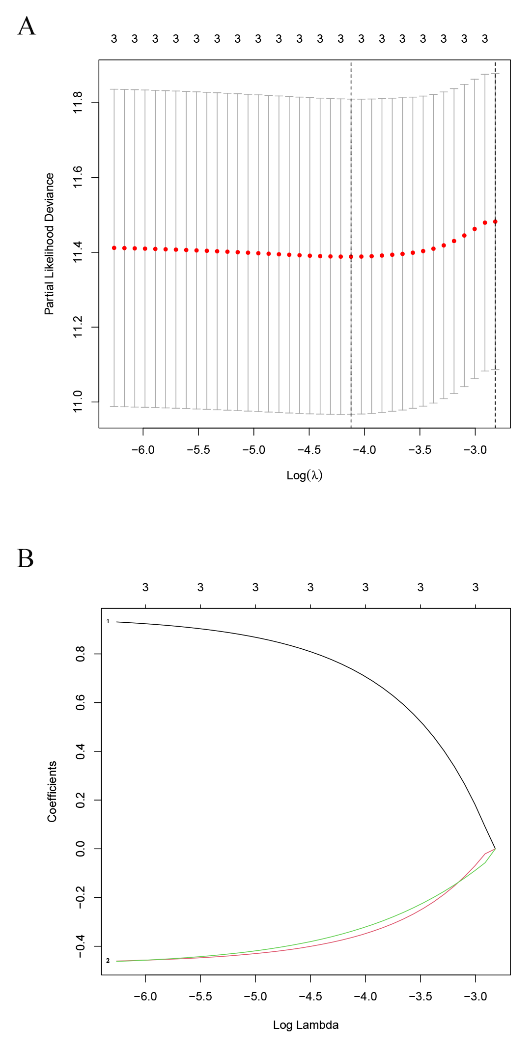


**Figure S2. LASSO regression for prognostic model construction.**

(A) Ten-fold cross-validation for tuning the parameter lambda in the LASSO Cox regression model. The dotted line indicates the optimal lambda value. (B) LASSO coefficient profiles of candidate genes as a function of the log(lambda) value. Three genes were selected at the optimal lambda.


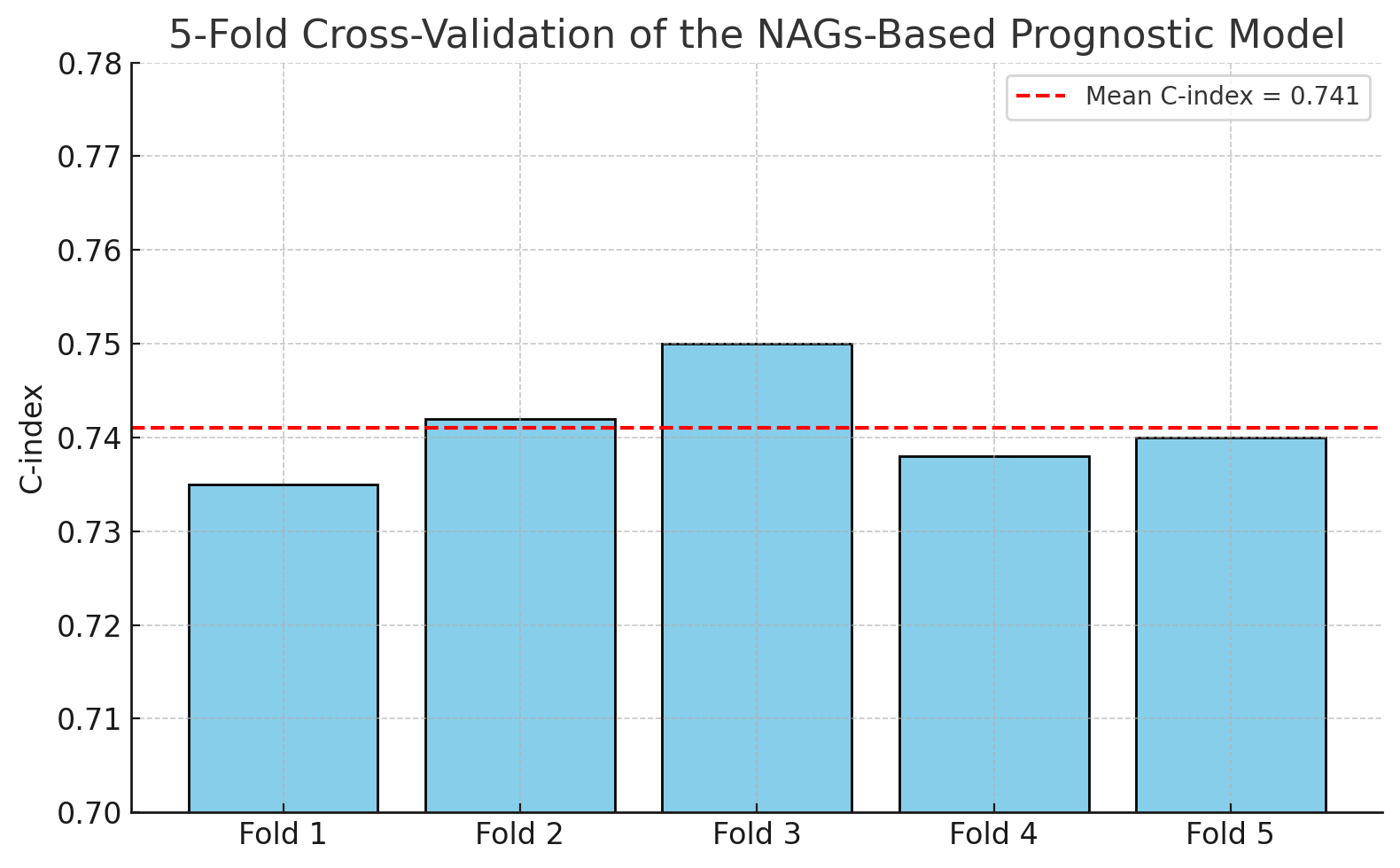


**Figure S3. 5-Fold Cross-Validation of the NAGS-Based Prognostic Model in the TCGA Cohort.**

Bar plot showing the concordance index (C-index) obtained from 5-fold cross-validation to assess the predictive robustness of the NAGS-based risk model. Each bar represents the C-index from one validation fold, and the red dashed line indicates the mean C-index value (0.741) across all folds.


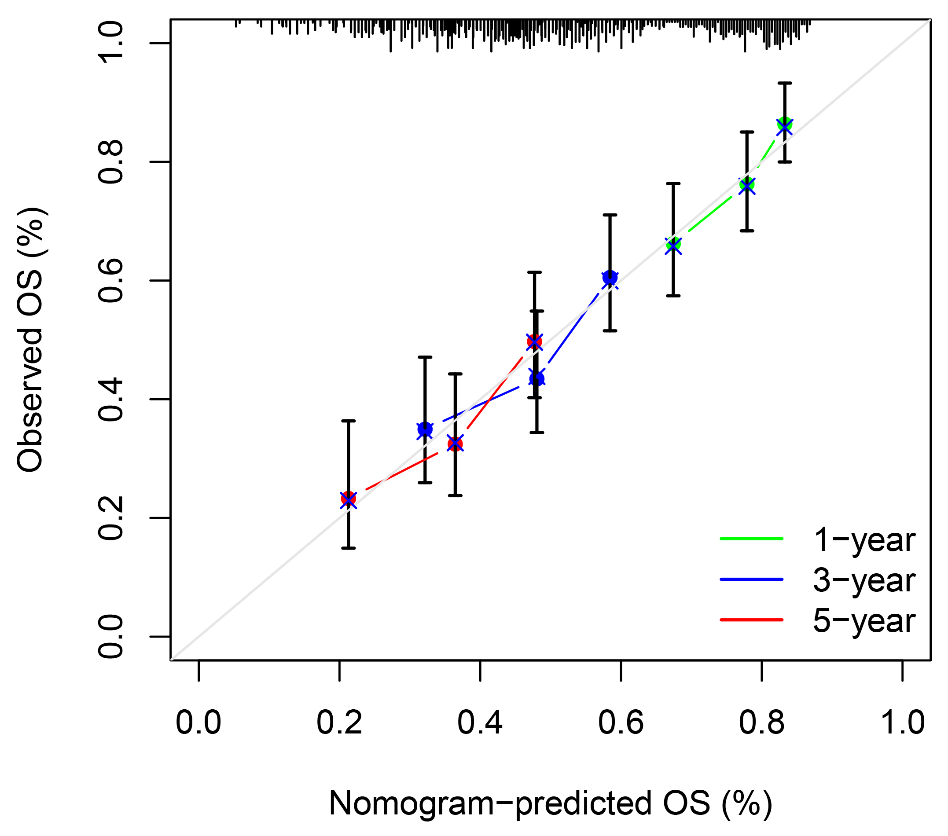


**Figure S4. Calibration plot of the nomogram for predicting overall survival.**

Calibration curves for 1-, 3-, and 5-year overall survival (OS) demonstrate strong agreement between nomogram-predicted and observed outcomes, indicating good predictive accuracy.

**
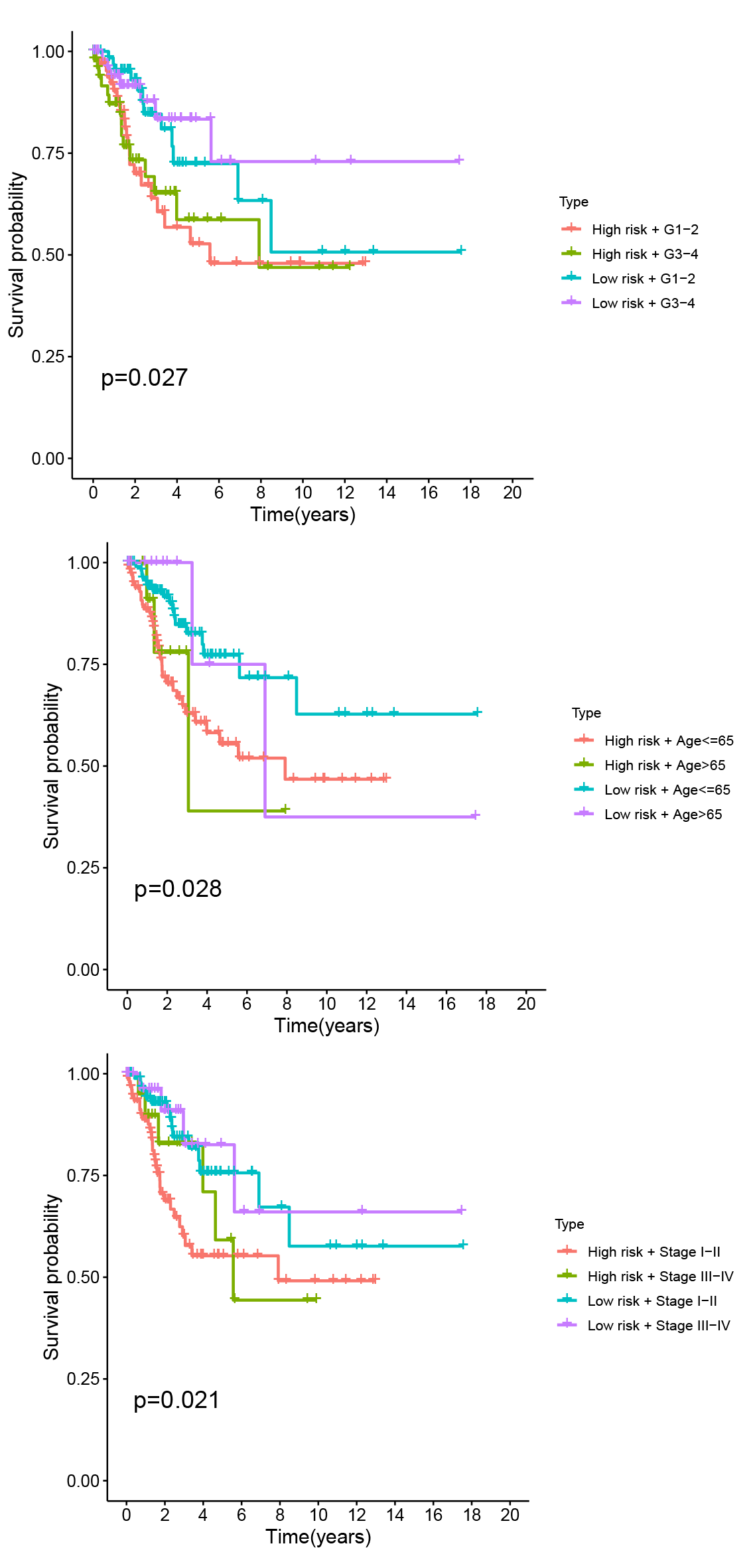
**

**Figure S5. Stratified survival analysis of the NAGS-based risk model.**

Kaplan–Meier curves showing overall survival in cervical cancer patients stratified by risk score and (top) tumor grade, (middle) age, and (bottom) clinical stage.

**
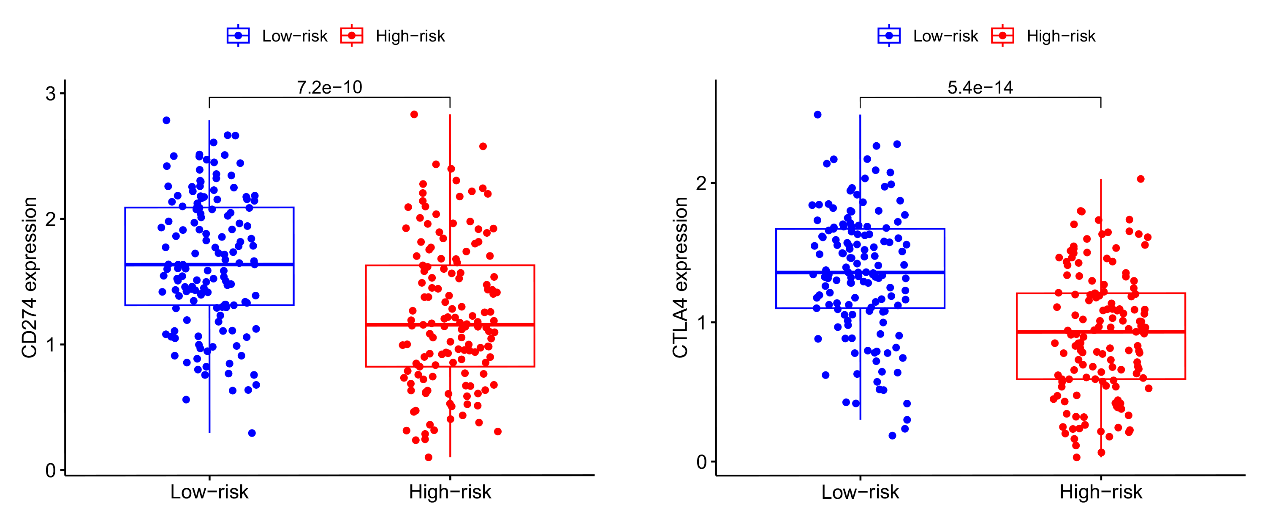
**

**Figure S6. Expression of immune checkpoint molecules CD274 (PD-L1) and CTLA4 between risk groups.**

**
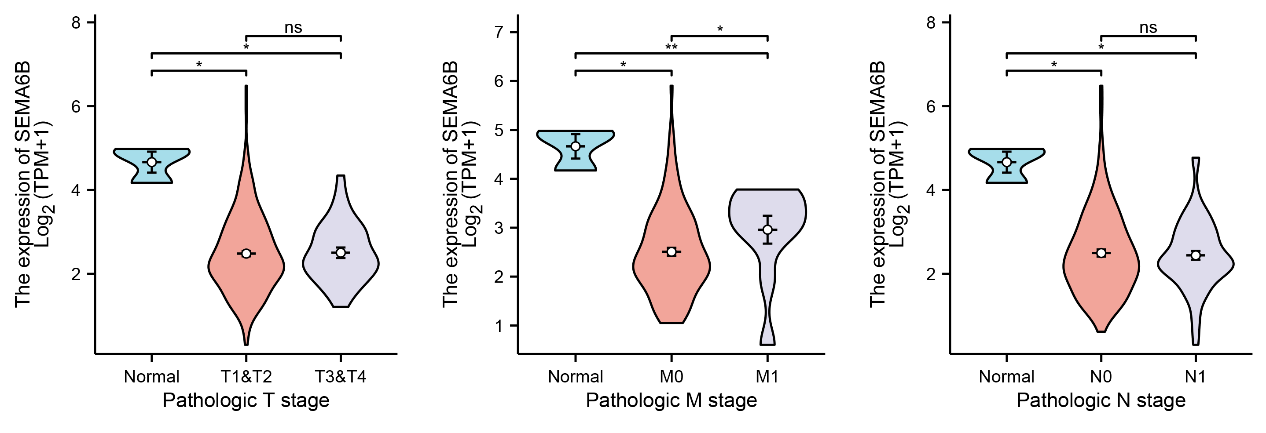
**

**Figure S7. SEMA6B expression across pathological stages in cervical cancer.**

Violin plots showing SEMA6B expression levels in normal tissue and tumor samples stratified by (left) T stage, (middle) M stage, and (right) N stage. SEMA6B expression was significantly downregulated in tumors compared to normal tissues across all stages. A slight upregulation trend was observed in M1 stage compared to M0. *, p < 0.05; **, p < 0.01; and ***, p < 0.001, not significant.


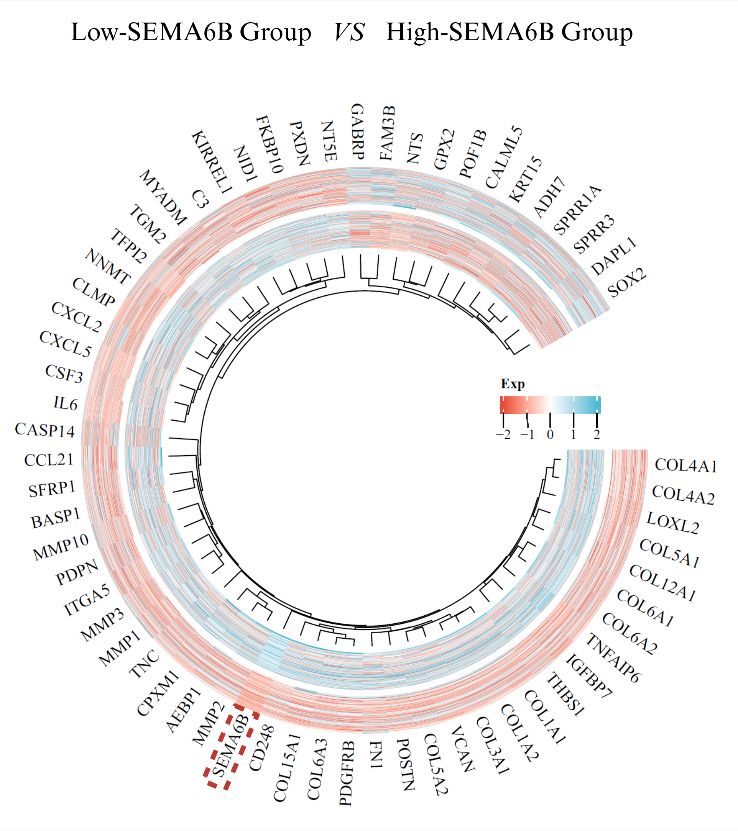


**Figure S8. Differentially expressed genes (DEGs) between high- and low-SEMA6B expression groups.**

Circular heatmap displaying 71 DEGs identified between the high- and low-SEMA6B subgroups (|log₂FC| ≥ 2, p < 0.05).


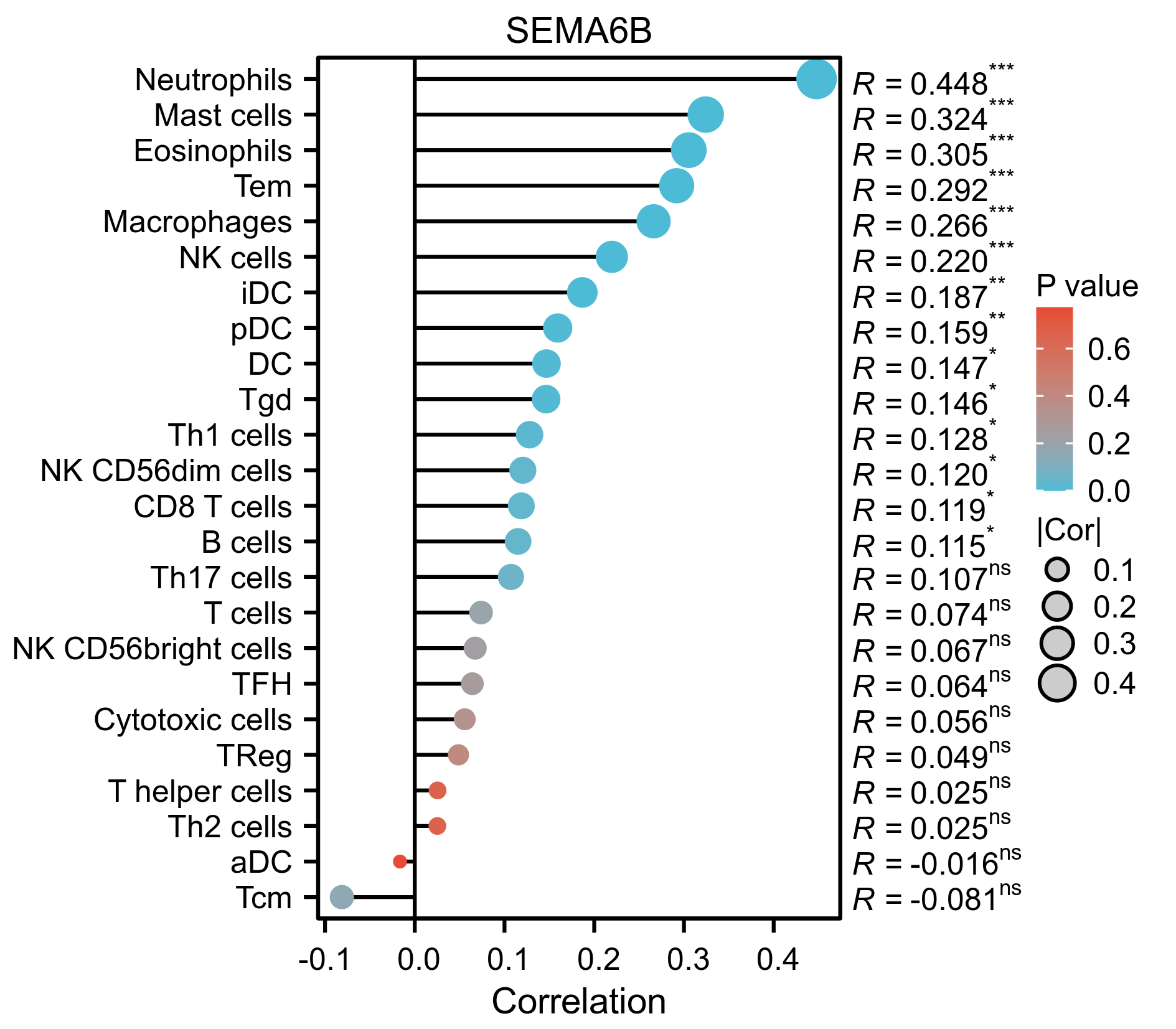


**Figure S9. Correlation Between SEMA6B Expression and Immune Cell Infiltration Estimated by ssGSEA.**

Bubble plot showing Spearman correlation between SEMA6B expression and 28 immune cell subsets using the single-sample Gene Set Enrichment Analysis (ssGSEA) algorithm in the TCGA cervical cancer cohort. Bubble size represents the absolute correlation coefficient (|Cor|), and bubble color indicates the p-value scale (blue = significant; red = non-significant).

**Table S1. Hazard Ratios and Confidence Intervals for Prognostic Genes Included in the NAGS-Based Risk Model**

| Gene | HR | 95% CI (Lower) | 95% CI (Upper) | p-value |
| --- | --- | --- | --- | --- |
| SEMA6B | 2.04417 | 1.07878 | 3.87347 | 0.02834 |
| CSF2RB | 0.561 | 0.32328 | 0.97351 | 0.03984 |
| IRF4 | 0.54591 | 0.30907 | 0.96422 | 0.03703 |

Hazard ratios (HR), 95% confidence intervals (CI).
